# Protecting the health of the most vulnerable in the overlooked Democratic Republic of Congo crisis

**DOI:** 10.1002/hsr2.70011

**Published:** 2024-08-20

**Authors:** Sandra S. Phiri, Nsikakabasi S. George, Lucky Iseghehi

**Affiliations:** ^1^ Youth On Board Organization (YOBO) Blantyre Malawi; ^2^ Institute of Public Health, Faculty of Health Sciences, Jagiellonian University Medical College Krakow Poland; ^3^ School of Medicine and Population Health, The University of Sheffield Sheffield UK; ^4^ eHealth Africa Kano State Nigeria

**Keywords:** displaced people, DR Congo, food insecurity, humanitarian crisis, social determinants of health

## Abstract

**Background and Aim:**

The Democratic Republic of Congo (DRC) is facing a humanitarian crisis due to prolonged conflicts, human rights abuses, and gender‐based violence. This perspective article aims to discuss the challenges faced by vulnerable populations in the DRC, including the impact of interventions and broader humanitarian strategies.

**Methods:**

A search on Google Scholar was carried out to identify relevant journal articles. Additionally, relevant reports and data on the DR Congo crisis were sourced from the websites of international and humanitarian organizations including the United Nations High Commissioner for Refugees, World Health Organization, United Nations Office for the Coordination of Humanitarian Affairs, and the Norwegian Refugee Council.

**Results:**

The crisis has led to 6.1 million displaced individuals and 1 million seeking asylum in neighboring countries. Insecurity and displacement further exacerbate the crisis, exacerbating health issues and malnutrition, particularly among women and children. The deterioration of water, sanitation, and hygiene infrastructure in the region facilitates the spread of infectious diseases. The UN has appealed for $8.3 billion in humanitarian assistance in West and Central Africa, including DRC. However, more attention and efforts are needed to curb the menace of the conflict.

**Conclusions:**

Addressing root causes like political volatility, economic disparity, and social unrest is crucial for sustained health protection. Tailoring humanitarian assistance to the context of conflict is essential, prioritizing mental health support and psychosocial services. Improving access to healthcare is crucial. Addressing food insecurity is essential, involving targeted food assistance programs, improving agricultural practices, and establishing income‐generating activities. Sustaining international assistance and investments are needed to address the health needs of the most vulnerable populations.

## 1 INTRODUCTION

Billions of people around the world reside in conflict‐ridden facing violence, malnutrition and diseases, with conflicts exerting an enormous influence on the social determinants of health.^1^, ^2^ An estimated 300 million people globally will require humanitarian assistance in 2024 stemming from the surge of climate emergencies, conflicts, disasters, and other phenomenon, and about 22% of these population will be in West and Central Africa.^1^

The Democratic Republic of Congo (DRC), often overshadowed by other global events, has long been grappling with a humanitarian crisis that has severe implications for the well‐being of the population especially the most vulnerable populations, including but not limited to women and children, older adults, people with disabilities, and chronic illnesses. According to the International Rescue Committee, the crisis in the DRC which is multifaceted with deep‐rooted causes, including resource exploitation, political instability, ethnic rivalries, armed militias, and regional interests is escalating, as the country remained on the Watchlist top 10 crises for 2023.^3^ The conflict in the east of the country is deepening economic challenges and disease outbreaks, with millions of people already in need of humanitarian aid.^4^ The crisis in the DRC is one of the most intricate humanitarian emergencies globally. The country has experienced prolonged conflicts, numerous human rights abuses, and severe cases of gender‐based violence, resulting in an unprecedented number of people in need of protection, vulnerable to harm, and at risk. As a result, 6.1 million individuals have been displaced within the country, while 1 million have been compelled to seek asylum in various African nations.^4^ Simultaneously, the DRC accommodates over 500,000 refugees from neighboring countries.^4^ The situation, however, is likely to worsen in the coming year, and there is a need for urgent action to address the root causes of the crisis. As conflict and instability persist in the region, the health and well‐being of these marginalized groups are at significant risk.

## 2 CURRENT CHALLENGES

From a humanitarian standpoint, several issues challenges exacerbate the crisis, necessitating urgent attention and comprehensive solutions. The prolonged conflict placed immense pressure on the already fragile healthcare system in the DRC. Medical facilities, such as hospitals and clinics are often targets of violence, leading to the destruction of medical facilities and hindering the delivery of essential healthcare services.^5^ Healthcare workers frequently face assault and sometimes are compelled to provide basic care without prioritizing medical ethics to protect their own lives.^6^ The limited availability of healthcare workers with only 0.28 medical professionals per 10,000 individuals, significantly fall short of global target of 22.8 healthcare workers per 10,000 exacerbates the situation, making it challenging to provide adequate medical care to individuals who require it.^7^ The DRC healthcare system is further compromised by inadequate logistic resources, shortage of diagnostic facilities, a dearth of personal protective equipment, and divergent stakeholder interests.

Insecurity and displacement hinder access to healthcare services, exacerbating health issues and malnutrition, particularly among women and children.^8^ DRC ranked third highest estimated number of global maternal deaths after India.^9^ Inadequate maternal and child health service provisions, including antenatal care, postnatal care, presence of competent carers during childbirth, and regular check‐ups for children account for this.^10^ Conflict‐induced displacement disrupts the continuity of healthcare services, leading to decreased access to crucial services including maternal care, vaccination, and treatment for chronic diseases.^11^ This is compounded by the deterioration of water, sanitation, and hygiene (WASH) infrastructure in the region which facilitates the spread of infectious diseases. Waterborne diseases like cholera and vector‐borne diseases such as malaria, Ebola, meningitis, and bubonic fever have become rampant due to poor sanitation, limited access to safe drinking water, and the displacement of populations, thus, further compromising the health and nutritional status of vulnerable populations.^12^ Poor WASH conditions contribute to the spread of diseases. The conflict, displacement, and economic challenges also contribute to severe food insecurity in the DRC, leading to malnutrition, especially among vulnerable groups.^12^ More so, vulnerable groups face increased risks of violence, exploitation, and neglect, leading to worsening health outcomes.

## 3 PROSPECTS AND RECOMMENDATIONS

Coordinated efforts from humanitarian organizations and the international community have in the last decade responded to crisis in DRC with provided support including but not limited to emergency medical and nutritional supplies, funding, technical support and expertise, and volunteer time to address the critical needs of the population. The Congo Humanitarian Fund received about US $46 million in funding in 2022 from the international community.^13^ Significant contributions and support have also been received from the African Union and member states, the United States (US), the European Union (EU), the Government of Japan, and other member states of the United Nations (UN) to provide life‐saving assistance to the displaced people in DRC.^14^, ^15^, ^16^ These supports have been tremendous in ensuring access to essential health services and strengthening collaborations with these communities. Furthermore, an appeal of US $8.3 billion have been made by the UN and other organizations in 2024 to support humanitarian assistance in West and Central Africa including DRC.^1^ However, to ensure sustained impact, more attention and efforts are still needed to curb the menace of the conflict.

First, it is important to address the ongoing conflict in the DRC. The conflict has been a major driver of the humanitarian crisis in the country, and it is important to find a peaceful resolution to the conflict. This includes supporting peace negotiations and providing humanitarian assistance to those affected by the conflict. Addressing the root causes of the conflict, such as political volatility, economic disparity, and social unrest is crucial for sustained health protection. Diplomatic efforts and international collaboration especially in the African Union can contribute to fostering stability and security in the region. As a key mitigation strategy, it is sacrosanct to tailor humanitarian assistance to the context of conflict, considering the specific needs and risks faced by vulnerable groups, prioritizing mental health support and psychosocial services for those affected by the crisis is essential to address the long‐term impact of trauma and displacement. This involves coordinating with local actors to ensure the safety and well‐being of the population, strengthening protection mechanisms, including gender‐based violence prevention, child protection, support for people with disabilities, and creating safe spaces for vulnerable individuals to access essential services.

Another recommendation for protecting the health of the most vulnerable in the DRC is to improve access to health care. The DRC has one of the lowest healthcare coverage rates in the world, and many people do not have access to basic healthcare services. It is important to invest in healthcare infrastructure and to provide access to basic healthcare services to the most vulnerable. This includes providing access to vaccinations, maternal and child health services, and treatment for infectious diseases such as malaria and tuberculosis, investing in the reconstruction of healthcare facilities, providing training for healthcare workers, and establishing a secure work environment and atmosphere for medical personnel and healthcare providers drawing on humanitarian principles. In this transient context, establishing and deploying mobile health units can help reach remote areas with health services. Furthermore, there is a need for intensive emergency care training and infrastructure, which would enable care providers to respond effectively and support the most vulnerable during the crisis. The WASH challenges in vulnerable populations can be mitigated through investment in WASH infrastructure, promotion of hygiene education, and ensuring access to clean water, which are crucial steps in reducing waterborne diseases and enhancing overall health. Similarly, launching community‐based education programs focusing on nutrition, hygiene, and health can empower communities with knowledge to make informed decisions, thereby improving their well‐being. More so, it is essential to engage communities in the planning and implementation of WASH projects to ensure sustainability and community ownership. Additionally, conducting hygiene promotion campaigns can raise awareness about the significance of clean water, proper sanitation, and good hygiene practices in preventing diseases.

Addressing the food insecurity is an essential driver for protecting the health of the most vulnerable. Expanding recommendations for addressing nutrition and humanitarian challenges in DR Congo involving the implementation of targeted food assistance programs, improving agricultural practices, and establishing income‐generating activities will enhance food security. Integrated nutrition programs addressing both acute and chronic malnutrition, involving local communities in their design and implementation, can significantly contribute to addressing food insecurity. More so, promoting sustainable agricultural practices such as crop diversification and agroforestry, along with supporting income‐generating initiatives, particularly for women, can enhance agricultural resilience and empower households economically, leading to increased access to food and essential services. In terms of maternal and child health services, education and skill‐building programs through community workshops on nutrition, health, and hygiene education, as well as empowerment programs for women and youth, play a crucial role. By equipping individuals with knowledge and skills, these initiatives aim to contribute to household income, improve community well‐being, and build resilience against multifaceted challenges faced by vulnerable populations in DR Congo. Through a combination of nutrition‐specific interventions and broader humanitarian strategies, the goal to empower communities, enhance resilience, and improve overall health outcomes in the region is feasible.

## 4 CONCLUSION

Protecting the health of the most vulnerable populations in the DRC crisis is a moral imperative and a public health priority. Improving access to healthcare in the DR Congo will require a multi‐faceted approach that involves strengthening local healthcare systems, increasing access to medical supplies and personnel, and addressing the social determinants of health that disproportionately affect vulnerable populations. To effectively address the health needs of the most vulnerable, there is a crucial need for sustained international assistance and investments. The international community must elevate the issue of the DR Congo crisis and prioritize the protection of the most vulnerable populations. By doing so, we can work towards ensuring that all individuals in the country have the opportunity to lead healthy and fulfilling lives, regardless of their circumstances.

## AUTHOR CONTRIBUTIONS

**Sandra Salomy Phiri**: Conceptualization; data curation; formal analysis; writing—original draft; writing—review and editing. **Nsikakabasi Samuel George**: Conceptualization; writing—original draft; writing—review and editing; formal analysis; data curation. **Lucky Iseghehi**: Writing—original draft; formal analysis; data curation.

## CONFLICT OF INTEREST STATEMENT

The authors declare no conflict of interest.

## TRANSPARENCY STATEMENT

The lead author Sandra Salomy Phiri affirms that this manuscript is an honest, accurate, and transparent account of the study being reported; that no important aspects of the study have been omitted; and that any discrepancies from the study as planned (and, if relevant, registered) have been explained.

## Data Availability

Data sharing is not applicable to this article as no datasets were generated or analyzed during the current study. The authors confirm that the data supporting the findings of this study are available within the article.
